# Methodology and data for quantifying storm erosion potential considering sea level rise

**DOI:** 10.1016/j.dib.2024.110685

**Published:** 2024-06-26

**Authors:** Audrey C. Fanning, Matthew S. Janssen, Laura Lemke, Jon K. Miller

**Affiliations:** a Davidson Laboratory, Stevens Institute of Technology, Hoboken, NJ 07030, USA; b Guy Carpenter & Company LLC, 1166 Avenue of the Americas, New York, NY 10036, USA

**Keywords:** Storm intensity, Climatology, Climate change, Coastal protection, Natural hazards, Storm damage reduction, Coastal erosion

## Abstract

This dataset quantifies storm intensity of approximately 130 unique historical storms along the New Jersey coastline from 1980 to 2014 for three separate sea level conditions. Namely, (1) as observed in the historical record; (2) detrended to 1997 mean sea level and (3) adjusted to the 2050 and 2100 sea level rise scenarios presented in the International Panel on Climate Change's (IPCC) Sixth Assessment Report (AR6). Projected sea level scenarios are adjusted to include local vertical land movement. Storm intensity is quantified in terms of erosion potential, considering the combination of total water level, wave heights, and storm duration. The observational dataset includes both tropical and extratropical storms and quantifies both the cumulative (duration) and peak (single hour) storm intensity for each storm and sea level rise (SLR) condition. Additionally, hourly time series of wave characteristics and water levels are provided at 13 locations along the New Jersey coast, facilitating hydrodynamic forcing of nearshore models. The dataset provides the means and methods to directly compare historical storms under future SLR conditions.

Specifications Table

**Table:** 

Subject	Climatology
Specific subject area	Storm erosion potential climatology for historical storms observed along the New Jersey, USA coastline subject to the IPCC's sea level rise scenarios
Type of data	Raw, Analysed
Data collection	Water level data was downloaded from New York Harbor Observing and Prediction System (NYHOPS) hindcasts. Wave data was downloaded from the U.S. Army Corps of Engineers Wave Information Study (WIS). The water level and wave height data sets were combined in MATLAB to create the observed storm climatology. The baseline climatology was created by detrending the observed climatology to 1997 MSL to eliminate the observed SLR signal. Future climatologies were created by adding estimates of sea level rise to the baseline data. All data was created using MATLAB software.
Data source location	The segment boundaries and offshore extraction points for each segment are listed below: Segment NJ1: Sandy Hook to Sea Bright (40.42°N, -73.83°W) Segment NJ2: Sea Bright to Long Branch (40.33°N, -73.83°W) Segment NJ3: Long Branch to Shark River Inlet (40.25°N, -73.83°W) Segment NJ4: Shark River Inlet to Manasquan Inlet (40.08°N, -73.83°W) Segment NJ5: Manasquan Inlet to Barnegat Inlet (39.92°N, -73.92°W) Segment NJ6: Barnegat Inlet to Little Egg Inlet (39.50°N, -74.00°W) Segment NJ7: Little Egg Inlet to Absecon Inlet (39.33°N, -74.17°W) Segment NJ8: Absecon Inlet to Great Egg Harbor Inlet (39.17°N, -74.33°W) Segment NJ9: Great Egg Harbor Inlet to Corson Inlet (39.17°N, -74.42°W) Segment NJ10: Corson's Inlet to Townsends Inlet (39.08°N, -74.50°W) Segment NJ11: Townsends Inlet to Hereford Inlet (39.00°N, -74.50°W) Segment NJ12: Hereford Inlet to Cape May Inlet (38.92°N, -74.58°W) Segment NJ13: Cape May Inlet to Delaware Bay (38.83°N, -74.75°W)
Data accessibility	Repository name: Mendeley Data Data identification number: 10.17632/8ffns74X74.1 Direct URL to data: http://dx.doi.org/10.17632/8ffns74X74.1 Instructions for accessing these data: N/A
Related research article	None

## 1 Value of the Data

• The data quantifies storm intensity by erosion potential for historical storms under three scenarios: (1) as occurred in the historical record; (2) detrended to remove the observed influence of sea level rise; (3) the observed storm with the added effects of the IPCC sea level rise scenarios for 2050 and 2100. The Storm Erosion Index and its derivatives were used to evaluate erosion potential through the combination of the three primary drives of erosion: water levels, wave heights, and storm duration. The Storm Erosion Index (SEI) categorized the cumulative erosion potential of storms while the Peak Erosion Index (PEI) categorized the peak erosion potential.
• Approximately 130 unique storms are evaluated in the dataset. The dataset contains bulk statistics of peak water levels and wave heights along with measures of cumulative (SEI) and peak (PEI) erosion potential for each storm. Hourly time series of wave characteristics and water levels for the duration of record (1980–2014) along the New Jersey coast are also included.
• This data may be used to evaluate potential storm impacts under historical and projected sea level scenarios. Bulk statistics are provided for analysis of the authors’ storm categorization while time series are provided for independent analysis and forcings of numerical models.
• The distinguishing feature is the analysis of erosion potential through Storm Erosion Index does not explicitly differentiate between tropical and extratropical storms, allowing direct comparison of all storms and particularly atypical storms.
• Use of the Storm Erosion Index is advantageous when evaluating morphological impacts. This dataset specifically has value in evaluating the impact of SLR on coastal storm erosion potential.

## 2 Background

This dataset presents a suite of historical storms projected into future sea level rise scenarios. Lemke and Miller [1] developed a 34-year climatology capable of identifying spatial and temporal erosion trends within New Jersey, USA through the evaluation of storm erosion potential. This climatology was detrended to remove the observational sea level rise signal and then projected to 2050 and 2100 sea levels based on the International Panel on Climate Change's (IPCC) Sixth Assessment Report (AR6) [2]. Like Lemke and Miller, the Storm Erosion Index and its derivatives were used to quantify cumulative and peak storm erosion potential. This dataset was created with the intent of quantifying the impact that sea level rise will have on storm erosion potential in New Jersey. However, the methodology outlined can be used to develop similar climatologies to investigate the impact of SLR on storm erosion potential within other geographical regions.

## 3 Data Description

This data quantifies storm intensity through erosion potential. Erosion potential is defined using the Storm Erosion Index [3] which combines the three main drivers of coastal erosion; elevated water levels, wave heights, and duration in a physically meaningful way. The dataset allows direct comparison of tropical and extratropical storms based on an observational 34-year record (1980–2014). Reported metrics include cumulative (Storm Erosion Index, abbreviated as SEI) and peak (Peak Erosional Intensity, abbreviated as PEI) measures of storm intensity. To extend the usefulness of the original (herein referred to as the ‘observational’) climatology [1], the storm time series are modified herein to include various sea level rise scenarios consistent with the most recent AR6 IPCC projections [2]. The dataset will be of use to coastal engineers, scientists, and managers interested in quantifying morphological changes in historical context and accounting for climate change. The dataset provides a method to compare historical storm intensities of tropical and extra-tropical storms.

In total, there are 12 climate scenarios contained in this data set. These scenarios include the observational climatology presented in Lemke and Miller [1], the baseline climatology (the observed record detrended to remove historical sea level rise), and the baseline record including future sea level rise. Sea level rise scenarios are published by the IPCC and include: SSP1-1.9 2050, SSP1-2.6 2050, SSP2-4.5 2050, SSP3-7.0 2050, SSP5-8.5 2050, SSP1-1.9 2100, SSP1-2.6 2100, SSP2-4.5 2100, SSP3-7.0 2100, and SSP5-8.5 2100. Each climate scenario has 26 associated .csv files, one raw data file and one bulk statistic data file for each of the thirteen shoreline segments, resulting in 312 .csv files in total. The raw data file contains the water level (NYHOPS) and wave height (WIS Hindcast) times series from 1980 to 2014 utilized to develop the SEI climatology [1]. Variables include offshore water level (WL), offshore significant wave height (Hs), peak period (T), mean wave direction (MWD), the wave angle with respect to shore normal (ThetaNorm), breaking wave height (Hb), the raw instantaneous erosion intensity (IEI), and the processed instantaneous erosion intensity (IEI*).

Lemke and Miller [1] outlined various thresholds (i.e., water level, breaking wave height, steepness) to determine if hydrodynamic conditions within a timestep should be classified as a storm condition. If thresholds are exceeded, the timestep is considered a storm event and the calculated IEI will be included the storm's cumulative SEI calculation. Thus, the “IEI” variable is unprocessed and calculated for all timesteps which do not meet the designated storm criteria. If one were to index the IEI variable using the start and end dates of a storm, the calculated SEI may not represent the SEI value presented in the bulk statistic file. However, the “IEI*” variable considers the storm criteria presented in this paper and Lemke and Miller [1] giving an averaged IEI value for timesteps which drop out a storm. Indexing the IEI* variable with the start and end dates of a storm will always produce an SEI value which matches the one presented in the bulk statistic file. Both values are presented for transparency. Differences in IEI and IEI* are typically around lower intensity events (low wave heights/water levels or wave steepness).

The second .csv file affiliated with each shoreline segment and scenario combination contains the bulk statistics of identified storms. Each row of data represents a singular storm with each column containing a different storm statistic. The statistics included are the storm's start date and time (StormStart), end date and time (StormEnd), duration (Duration), Storm Erosion Index (SEI), Storm Erosion Index category (SEIcat), Peak Erosion Index (PEI), Peak Erosion Index category (PEIcat), maximum water level (MaxWL), maximum breaking wave height (MaxHb), state averaged SEI-based and PEI-based return periods and average probabilities (SEITrState, SEIAnnualProbState, PEITrState & PEIAnnualProbState), and the segment specific SEI-based and PEI-based return periods and average probabilities (SEITrSeg, SEIAnnualProbSeg, PEITrSeg & PEIAnnualProbSeg). Table 1 lists each scenario along with the naming structure of the corresponding time series and bulk statistic files while Table 2 lists variables, their units, and the name of the file which the variable is contained in.

**Table 1:** Climate scenarios contained in the dataset along with corresponding file name for the time series and bulk statistic .csv files.

Scenario	Time Series .csv	Bulk Statistic .csv
Lemke and Miller (2020)	NJ#_RawData_Observed.csv	NJ#_BulkStats_Observed.csv
Baseline	NJ#_RawData_Baseline.csv	NJ#_BulkStats_Baseline.csv
SSP1-1.9 2050	NJ#_RawData_SSP119_2050.csv	NJ#_BulkStats_ SSP119_2050.csv
SSP1-2.6 2050	NJ#_RawData_SSP126_2050.csv	NJ#_BulkStats_ SSP126_2050.csv
SSP2-4.5 2050	NJ#_RawData_SSP245_2050.csv	NJ#_BulkStats_ SSP245_2050.csv
SSP3-7.0 2050	NJ#_RawData_SSP370_2050.csv	NJ#_BulkStats_ SSP370_2050.csv
SSP5–8.5 2050	NJ#_RawData_SSP585_2050.csv	NJ#_BulkStats_ SSP585_2050.csv
SSP1-1.9 2100	NJ#_RawData_SSP119_2100.csv	NJ#_BulkStats_ SSP119_2100.csv
SSP1-2.6 2100	NJ#_RawData_SSP126_2100.csv	NJ#_BulkStats_ SSP126_2100.csv
SSP2-4.5 2100	NJ#_RawData_SSP245_2100.csv	NJ#_BulkStats_ SSP245_2100.csv
SSP3-7.0 2100	NJ#_RawData_SSP370_2100.csv	NJ#_BulkStats_ SSP370_2100.csv
SSP5-8.5 2100	NJ#_RawData_SSP585_2100.csv	NJ#_BulkStats_ SSP585_2100.csv

# represents Shoreline Segments 1–13.

**Table 2:** Variables with unit of measure and the file type in which the variable is contained.

Variable	Unit	File Type
WL, Hs, Hb	Meters	Time Series
IEI, IEI*	N/a	Time Series
T	Seconds	Time Series
MWD, ThetaNorm	Degrees	Time Series
SEI, PEI, SEICat, PEICat	N/a	Bulk Statistic
Duration	Hours	Bulk Statistic
SEITrState, PEITrState, SEITrSect, PEITrSect	Years	Bulk Statistic
SEIAnnualProbState, PEIAnnualProbState, SEIAnnualProbSect, PEIAnnualProbSect	1 / Years	Bulk Statistic
MaxWL, MaxHb	Meters	Bulk Statistic

## 4 Experimental Design, Materials and Methods

### 4.1 Storm erosion index

Miller and Livermont [3] introduced the Instantaneous Erosion Intensity (IEI) (Eq. 1) as a representative measure of storm intensity at a given point in time further defining the Storm Erosion Index (SEI) as the sum of IEI over a storm's duration (t_d_) (Eq. 2) and the Peak Erosion Intensity (PEI) (Eq. 3) as the maximum value of IEI over a storm's duration. A time-varying form of the well-known Bruun Rule [4], the Storm Erosion Index and its derivatives combine the three primary drivers of coastal erosion (wave height, total water level, and duration) into a physically meaningful parameter.

(1) $$IEI\left(t_{i}\right)=\;W_{*}\left(t_{i}\right)\left[\frac{0.068H_{b}\left(t_{i}\right)+S\left(t_{i}\right)}{B+1.28H_{b}\left(t_{i}\right)}\right]$$

(2) $$SEI=\;\sum_{t_{d}}\text{IEI}\left(t_{i}\right)$$

(3) $$PEI=\;\text{max}\left(\left\{IEI\left(t_{i}\right)\right\}\;:i=1,\ldots ,t_{d}\right)$$

In the above equations, *H*_b_ is the breaking wave height, W* is the width of the active surfzone (approximated as the distance to the breakpoint), *B* is the berm height, *S* is the water level height above the mean sea level, and *t_i_* is a time index. *IEI* is measured in units of length and is representative of the theoretical landward progression of the equilibrium shoreline position (measured in the cross-shore) if storm conditions were to continue indefinitely. Despite its theoretical meaning, IEI does not directly represent shoreline change. Instead, the metric has been shown to correlate well with spatial and temporal variations in storm intensity; capturing peak and duration measures in a physically meaningful manner [5] and when combined with beach state, can predict the shoreline response, including magnitude of dune erosion [6,7].

Lemke and Miller [1] utilized the Storm Erosion Index to develop a storm erosion climatology for the New Jersey coast employing various thresholds and criteria for identifying and defining a storm. The following criteria were also employed in the creation of this dataset for consistency.

• A storm is defined as the period of time where either the breaking wave height exceeds the 95 % threshold, or the water level exceeds the 99.9 % threshold. Thresholds are defined by data contained in the period of study (1980–2014).
• Storm duration (t_d_) is defined as the total number of hours between the first and last exceedance of one of the storm thresholds. Two exceedances separated by less than 48 h are considered a part of the same storm.
• Only waves which are both directed onshore and erosional are considered in a storm's SEI calculation. Timesteps with a wave angle greater than 90° with respect to shore normal or a wave steepness less than 0.2 drop out of the SEI calculation as these conditions are not considered erosional. Dropped out timesteps are replaced with an averaged IEI value.

### 4.2 Key assumptions

The objective of this dataset is to quantify the effect sea level rise will have on the storm erosion potential of historical storms observed along the coast of New Jersey. Consequently, the key assumptions are:

• The change in storm intensity (i.e., erosional potential) is solely due to the increase in mean sea level associated with appropriate IPCC scenario(s).
• All other climatic changes, particularly those affecting storm intensity (e.g., increased wave heights, duration) and storm frequency are neglected from the analysis.
• Adaption of the equilibrium beach profile to sea level rise (i.e. berm elevation) is neglected from the analysis.

Effectively this means the time series of significant wave height data used to create the climatology in Lemke and Miller [1] is held constant for this study in addition to the representative beach characteristics for New Jersey (median grain size of 0.4 mm and berm height of 2.5 m). Furthermore, the number of storms identified at each segment and the duration of each of these storms remained consistent with the observational climatology dataset. These key assumptions allow the increase in storm erosion potential to be attributed exclusively to increased sea levels.

While it is expected that the equilibrium profile will adapt to sea level rise, projections of sea level rise to 2050 are lower than historically observed interannual variations in MSL for which berm elevations have remained constant. This assumption is supported by the work of Houston [8], who found the current rates of nourishment can offset SLR induced erosion rates along the Florida coasts while maintaining historic berm elevations. By 2100, relative sea level rise rates will exceed observed interannual variations eliciting a profile response, most probably an increase in berm elevation, through natural or anthropogenic means.

The above assumptions provide a baseline or minimum increase in the coastal storm erosional hazard that can be expected in the forthcoming decades. This methodology can be used with any sea level rise projection to estimate future trends in the erosive potential of coastal storms.

### 4.3 Definition of tidal datums

The sea level rise projections published by the IPCC [2] and outlined in Section 4.6 are relative to the average global mean sea level from the years 1995–2014. Mean sea level (MSL = 0) as defined in the extracted NYHOPS water level data is relative to the 1983–2001 epoch. When comparing the mean water level of the NYHOPS data from 1995 to 2014 to MSL = 0, there was a negligible difference between the two values. Therefore, it was assumed that mean sea level as defined by the 1983–2001 epoch was equal to mean sea level from 1995 to 2014. This assumption allows MSL as defined in the NYHOPS data to be used as the base sea state for additional sea level.

### 4.4 Baseline data set

**Fig. 1:**
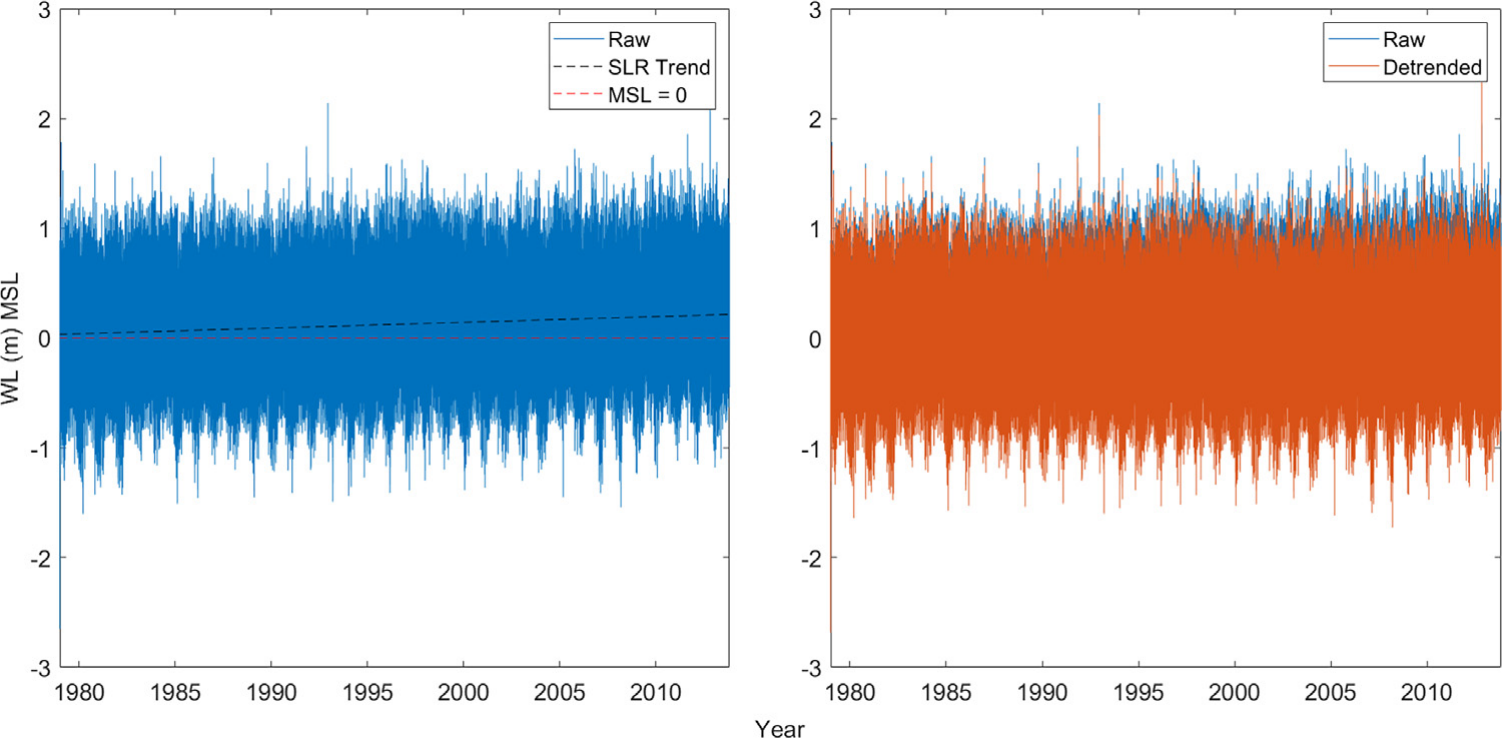
Identified sea level rise trend (black dashed line) for Shoreline Segment NJ1 compared to MSL = 0 (red dashed line) [left]. Detrended water level data (orange) overlaid on the raw water level data (blue) [right].(For interpretation of the references to color in this figure legend, the reader is referred to the web version of this article.)

The climatology data presented by Lemke and Miller [1] included the effects of local sea level rise during the duration of the 34-year period of study. Fig. 1 illustrates the observed sea level rise trend over the observational record for Shoreline Segment NJ1. To eliminate the sea level rise signal within the dataset, water level data for each segment was detrended to mean sea level. Removing the sea level rise signal removes importance of storm timing within the record and allows for all storms to be compared at an equivalent point in time. This detrended climatology is referred to as the “baseline climatology” and can be used to compare the effects of sea level rise scenarios on erosion potential.

### 4.5 *Global* vs *local sea level rise*

Global sea level rise is considered the average rate with which sea level is increasing worldwide. The IPCC affirms that global mean sea level (GMSL) has been increasing at a rate of 1.35 (0.78–1.95) mm per year in the instrumentation era (1901–1990) and a rate of 3.25 (2.88–3.61) in the satellite era (1993–2018) [2]. Due to subsidence, glacial rebound, and ocean currents, local (relative) sea level rise varies from the rate of global sea level rise. Specifically in New Jersey, the rate of local sea level rise, as measured by NOAA water level gauges stationed at Sandy Hook (8531680), Atlantic City (8534720), and Cape May (8536110), is higher than the rate of global sea level rise. A comparison between the local sea level rise rates recorded by NOAA's gauges and the NYHOPS hydrodynamic model is shown in Table 3. The observed rates of local sea level rise from the observational NOAA data are consistent to the extracted rates of local sea level rise in the NYHOPS dataset (Table 3). The median percent difference between the observed NOAA data and the modeled NYHOPS data was 2.5 % while the mean absolute error was 0.78 mm.

**Table 3:** Local sea level rise trends recorded by the NYHOPS hydrodynamic model and NOAAʼs water level gauges.

Segment	NOAA station	NOAA (mm/yr)	NYHOPS (mm/yr)	Δ (NOAA -NYHOPS)	Vertical Land Movement (mm/yr)
NJ1	Sandy Hook	5.45 (4.9–6.1)	5.20 (5.02–5.38)	0.25	−2.27 +/− 0.07
NJ2	Sandy Hook	5.45 (4.9–6.1)	6.62 (6.44–6.79)	−1.17	−2.27 +/− 0.07
NJ3	Sandy Hook	5.45 (4.9–6.1)	8.46 (8.29–8.64)	−3.01	−2.27 +/− 0.07
NJ4	Sandy Hook	5.45 (4.9–6.1)	6.40 (6.23–6.57)	−1.01	−2.27 +/− 0.07
NJ5	Sandy Hook	5.45 (4.9–6.1)	4.71 (4.55–4.88)	0.74	−2.27 +/− 0.07
NJ6	Atlantic City	5.30 (4.7–6.0)	5.20 (5.04–5.36)	0.10	−2.17 +/− 0.11
NJ7	Atlantic City	5.30 (4.7–6.0)	5.18 (5.02–5.34)	0.12	−2.17 +/− 0.11
NJ8	Atlantic City	5.30 (4.7–6.0)	5.22 (5.06–5.39)	0.08	−2.17 +/− 0.11
NJ9	Atlantic City	5.30 (4.7–6.0)	6.56 (6.39–6.73)	−1.26	−2.17 +/− 0.11
NJ10	Cape May	5.80 (5.2–6.4)	5.19 (5.02–5.35)	0.61	−2.10 +/− 0.25
NJ11	Cape May	5.80 (5.2–6.4)	5.17 (5.01–5.34)	0.63	−2.10 +/− 0.25
NJ12	Cape May	5.80 (5.2–6.4)	5.19 (5.02–5.36)	0.61	−2.10 +/− 0.25
NJ13	Cape May	5.80 (5.2–6.4)	5.19 (5.02–5.37)	0.61	−2.10 +/− 0.25

Measures of vertical land movement are the primary adjustment needed to apply global sea level rise projections to specific locations. Zervas*,* et al. [9] calculated the estimated vertical land movement from multiple NOAA tide gauges with at least 30 years of water level data. Vertical land movements for Sandy Hook, Atlantic City, and Cape May were outlined by Zervas, Gill and Sweet [9] and listed in Table 3.

### 4.6 Future sea level projections

Table 4 displays the IPCC sea level rise projections (2050, 2100) for the five Shared Socio-economic Pathways (SSP) scenarios relative to global mean sea level from 1995 to 2014. Totals are derived from contributions of thermal expansion, melting of the Greenland and Antarctica ice sheets, melting of glaciers, and changes in land–water storage.

**Table 4:** The projected increase in global mean sea level (GMSL) for the five SSP scenarios (SSP1-1.9, SSP1-2.6, SSP2-4.5, SSP3-7.0, and SSP5-8.5) in 2050 and 2100 relative to 1995–2014 GMSL as presented in AR6 of the IPCC.

	SSP1-1.9	SSP1-2.6	SSP2-4.5	SSP3-7.0	SSP5-8.5
Total (2050)	0.18 (0.15–0.23)	0.19 (0.16–0.25)	0.20 (0.17–0.26)	0.22 (0.18–0.27)	0.23 (0.20–0.29)
Total (2100)	0.38 (0.28–0.55)	0.44 (0.32–0.62)	0.56 (0.44–0.76)	0.68 (0.55–0.90)	0.77 (0.63–1.01)

As discussed in Section 4.5, rates of local sea level rise in New Jersey exceed rates of global sea level rise. To obtain an accurate projection of future sea level in New Jersey for 2050 and 2100, contributions of vertical land movement were included in future sea level estimates. The rates of vertical land movement summarized in Table 3 were added to the projected increases in global mean sea level outline in Table 4.

While the baseline data set is temporally independent over the 1980–2014 data period with respect to sea level, a “starting point” was needed to determine the contributions of vertical land movement to the overall increases in sea level. The midpoint (2005) of the relative sea level (1995–2014) period utilized by the IPCC was used as the “starting point” as a best estimate contributions of vertical land movement relative to the base sea level established by the IPCC. Eq. (4) outlines the method used to calculate the increase in local mean sea level (LSML) while Table 5 compiles the estimated total increase in sea level for 2050 and 2100 considering global and local contributions.

(4) LMSL(year)=SSPscenario(year)+VLM*(year−2005)

**Table 5:** Estimated increases in GMSL relative to 1995–2014 considering global increases in sea level presented by the IPCC and vertical land movement for the state of New Jersey.

	SSP1-1.9	SSP1-2.6	SSP2-4.5	SSP3-7.0	SSP5-8.5
2050					
NJ1–NJ5	0.282	0.292	0.302	0.322	0.332
NJ6–NJ9	0.278	0.288	0.298	0.318	0.328
NJ10–NJ13	0.275	0.285	0.295	0.315	0.325
2100					
NJ1–NJ5	0.596	0.656	0.776	0.896	0.986
NJ6–NJ9	0.586	0.646	0.766	0.886	0.976
NJ10–NJ13	0.580	0.640	0.760	0.880	0.970

To simulate increased sea levels, the values presented in Table 5 were added to each timestep within the detrended water level data effectively raising mean sea level. For example, 0.282 m was added to each hourly water level timestep for the 2050 SSP1-1.9 scenario to raise the mean sea level datum from 0 m to 0.282 m. The methodology outlined in Section 4.1 and assumptions outlined in Section 4.2 were applied to the sea level rise corrected data to create storm erosion climatology for each SSP scenario.

### 4.7 Storm return periods

Storm return periods (or annual probabilities) are independently calculated for peak and cumulative parameters. In the initial climatology work, Lemke and Miller [1] performed an Extreme Value Analysis (EVA) using a peaks-over-threshold analysis and the Generalized Pareto Distribution (GP). Despite careful considerations of the thresholds and optimizations techniques described in Palutikof*,* et al. [10] and Lang*,* et al. [11] Lemke and Miller acknowledged the poor resolution of the GP fit for events approaching or exceeding 1 % annual probabilities as a limitation of the length of their dataset.

To address the shortcoming in the length of the observational record, a bootstrap with replacement resampling technique was employed [12,13]. The only explicit assumption is the frequency and intensity of historical and future events remain statistically similar. Notably, this means increases in storm frequency, intensity or sea level rise (SLR) associated with climate change cannot be accounted for in this method. However, the process employs a robust interpolation, extrapolation and smoothing methodology which allows for a Monte-Carlo simulation to account for storms representative of the historical distributions, but not actually observed. A complete discussion can be found in Janssen [14].

Storm intensity values and return periods are provided in Table 6. Note, the analysis does not resolve return periods less 1 year. In other words, all storms returning index values less than or equal to 579 (SEI) and 46 (PEI) are assigned an annual probability of 99.9 % (and consequently a return period of 1.001). In the event sub-year return periods are of interest, the GP fit produced by Lemke and Miller [1] would be preferable.

**Table 6:** Estimated PEI and SEI values for given return periods for state-wide return periods.

Return period	Annual Prob	SEI		PEI	
		Value	ST. Dev.	Value	ST. Dev.
1.001	99.9 %	579	111	46	3
2	50 %	962	172	56	4
3	33 %	1181	202	61	5
4	25 %	1350	233	64	5
5	20 %	1504	264	67	5
6	17 %	1654	293	69	5
7	14 %	1805	319	71	5
8	13 %	1955	337	73	5
9	11 %	2101	349	75	5
10	10 %	2240	355	76	5
15	6.7 %	2778	368	84	6
20	5.0 %	3131	385	91	8
25	4.0 %	3379	403	98	11
30	3.3 %	3568	417	103	13
35	2.9 %	3718	428	108	15
40	2.5 %	3844	439	113	17
45	2.2 %	3951	446	117	19
50	2.0 %	4043	452	120	21
60	1.7 %	4199	464	127	24
70	1.4 %	4330	474	132	27
80	1.3 %	4439	482	137	29
90	1.1 %	4533	490	141	31
100	1.0 %	4614	495	144	33
150	0.667 %	4924	513	158	40
200	0.500 %	5136	529	167	44
250	0.400 %	5301	540	173	48
300	0.333 %	5444	552	179	50
350	0.286 %	5558	562	183	52
400	0.250 %	5663	571	187	54
500	0.200 %	5812	585	192	57
600	0.167 %	5988	598	199	59
700	0.143 %	6114	608	203	61
800	0.125 %	6209	615	207	63
900	0.111 %	6283	621	209	64
1000	0.100 %	6342	626	211	65

## Limitations

This methodology and data set only consider changes in erosion potential due to sea level rise. Climate change is also expected to influence the erosive potential of coastal storms through changes in storm intensity and storm frequency. There is little consensus on the quantification of these changes in literature leading the authors to neglect these variables from the analysis. Sea level rise is expected to influence multiple terms within the Storm Erosion Index (SEI) equation (Eqs. 1 and 2). The impact to storm surge (S) due to sea level rise is straightforward and easily quantified, however, the impact to berm height (B), width of the active surfzone (W*), and breaking wave height (Hb) are more complex. Therefore, the author's only quantified changes in storm erosion potential due to the increase in storm surge. The number of storms identified in the baseline climatology was held constant for the 2050 and 2100 scenarios essentially neglecting any changes in storm frequency. Within the Storm Erosion Index equations (Eqs. 1 and 2), only the surge (S) variable changed with SLR leading any increase in storm erosion potential to be solely attributed to increases in sea level.

## Ethics Statement

The authors have read and follow the ethical requirements for publication in Data in Brief and confirm that the current work does not involve human subjects, animal experiments, or any data collected from social media platforms.

## CRediT Author Statement

**Audrey C. Fanning:** Methodology, Formal analysis, Investigation, Data curation, Writing – Original Draft. **Matthew S. Janssen**: Conceptualization, Investigation, Writing – Original Draft. **Laura Lemke**: Software, Investigation, Data curation. **Jon K. Miller**: Writing – Review & editing, Supervision, Funding acquisition.

## Data Availability

Influence of Sea Level Rise on Storm Erosion Potential in New Jersey (Original data) (Mendeley Data).
